# A Specific Circulating MicroRNA Cluster Is Associated to Late Differential Cardiac Response to Doxorubicin-Induced Cardiotoxicity *In Vivo*

**DOI:** 10.1155/2018/8395651

**Published:** 2018-12-09

**Authors:** Clarissa Ruggeri, Sonia Gioffré, Mattia Chiesa, Marta Buzzetti, Giuseppina Milano, Alessandro Scopece, Laura Castiglioni, Marta Pontremoli, Luigi Sironi, Giulio Pompilio, Gualtiero I. Colombo, Yuri D'Alessandra

**Affiliations:** ^1^Immunology and Functional Genomics Unit, Centro Cardiologico Monzino IRCCS, Milan, Italy; ^2^Vascular Biology and Regenerative Medicine Unit, Centro Cardiologico Monzino IRCCS, Milan, Italy; ^3^Laboratory of Cardiovascular Research, Department of Surgery and Anesthesiology, University Hospital of Lausanne, Lausanne, Switzerland; ^4^Department of Pharmacological and Biomolecular Sciences, University of Milan, Milan, Italy; ^5^Unit of Cardio- and Cerebrovascular Research: Experimental Models and In Vivo Imaging, Centro Cardiologico Monzino IRCCS, Milan, Italy

## Abstract

**Background:**

Cardiotoxicity is a detrimental side effect of the anticancer drug doxorubicin (DOX), characterized by progressive heart dysfunction. Circulating microRNAs (miRNAs) are recognized as potential biomarkers of cardiac disease; thus, we aimed to investigate their association with late cardiotoxicity in an animal model of disease.

**Methods:**

Twenty C57BL/6 female mice were administered with 24 mg/kg cumulative dose of DOX or saline during 2 weeks, followed by a recovery period of one month (T42). Echocardiography was performed at baseline and at T42, and plasma samples were collected at T42. The selection of all miRNAs of interest was conducted by literature overview and by screening, followed by RT-qPCR validation*. Results.* The analysis of cardiac function at T42 evidenced five DOX-treated animals indistinguishable (NoTox) from controls (CTRLs), while four presented heart impairment (Tox). Our analyses identified eight dysfunction-associated plasma miRNAs. In particular, seven miRNAs were found downregulated in comparison to CTRLs, miR-1-3p, miR-122-5p, miR-127-3p, miR-133a-3p, miR-215-5p, miR-455-3-p, and miR-499a-5p. Conversely, miR-34a-5p showed increased levels in Tox plasma samples. Noteworthy, we determined a cluster composed of miR-1-3p, miR-34a-5p, miR-133a-3p, and miR-499a-5p that distinguished with high-accuracy Tox from NoTox mice.

**Conclusion:**

This is the first study indicating that, similarly to what is observed in patients, DOX-administered animals present a differential cardiac response to treatment. Moreover, our results indicate the presence of specific plasma miRNAs whose expression reflect the presence of cardiac dysfunction in response to drug-induced injury.

## 1. Introduction

Doxorubicin (DOX) is a chemotherapeutic agent belonging to the family of anthracycline drugs. It is currently used to treat several types of tumors, including breast cancer, leukemia, and lymphomas [1], but its administration is limited by cumulative dose-related cardiotoxicity [2]. Indeed, patients may experience cardiac symptoms varying from mild ventricular dysfunction to severe heart failure, possibly leading to heart transplantation [3]. According to recent guidelines by the American Society of Echocardiography and the European Association of Cardiovascular Imaging, cardiotoxicity is defined as a decline of left ventricular ejection fraction (LVEF) greater than 10 points [4]. It is very difficult to predict which patient will develop such an impairment, since it can occur either early during treatment or several years after administration. Thus, timely diagnosis of dysfunction onset is of paramount importance for those patients at risk of developing cardiac damage. LVEF echocardiographic monitoring, nuclear imaging, and, when indicated, endomyocardial biopsy are currently used to detect and monitor cardiotoxicity [5]. Unfortunately, these techniques share a limitation consisting in late diagnosis of heart impairment occurrence. In the last years, several circulating markers have been investigated with the aim of early assessment and prediction of cardiac dysfunction onset. Among them, cardiac troponins (cTn) and brain natriuretic peptide (BNP) possess the highest sensitivity and reliability [6], but there is still a need of additional tools for timely and precise assessment of myocardial injury severity and progression.

MicroRNAs (miRNAs) are small (22–24 nucleotides) endogenous noncoding RNAs that are involved in posttranscriptional regulation of gene expression via inhibition and/or degradation of target messenger RNAs (mRNAs). Their activity can influence multiple biological processes such as proliferation, differentiation, development, and cell death [7], and their dysregulation is accountable for various pathologies, including cancer and cardiac diseases [8]. In recent years, miRNAs emerged as possible specific biomarkers of several conditions because of their stable expression in almost all body fluids (e.g., blood, serum, plasma, urine, and saliva) and due to the development of accurate and quantitative techniques for their detection [9]. In particular, several groups investigated the potential use of circulating miRNAs in the diagnostic and/or prognostic setting of cardiovascular diseases, in order to formulate tailored therapeutic strategies [10]. A limited number of preclinical studies focused on the association of circulating miRNAs with DOX cardiotoxicity, mainly in the acute phase of treatment and for mechanistic insights rather than diagnostic purposes [11]. The present study aimed at investigating specific plasma miRNAs associated to late stages of DOX-induced cardiac damage in a mouse model.

## 2. Methods

### 2.1. Animal Model of Cardiotoxicity

C57BL/6 female mice (Charles River Laboratories), aged 10 weeks old, were administered with either saline (CTRL, *n* = 10) or doxorubicin hydrochloride (DOX, *n* = 10, cumulative dose 24 mg/kg, Sigma-Aldrich) by intraperitoneal injections (4 mg/kg) 3 times a week for 2 weeks, as previously reported [12]. Female mice were selected to mimic female breast cancer patients. The study was carried out in strict accordance with the recommendations of the Guide for the Care and Use of Laboratory Animals of the National Institutes of Health. The protocol was approved by the Committee on the Ethics of Animal Experiments of the University of Milan and by the Italian Ministry of Health (approval number 379/2015-PR). The mortality rate was 0% in CTRL animals, while one DOX-treated mouse died two weeks after the end of DOX treatment for unknown reasons and was removed from the study. Thus, the final number of DOX-treated mice was 9.

### 2.2. Cardiac Function Evaluation

Heart function was monitored by echocardiography using a Vevo 2100 high-resolution imaging system (VisualSonics) before treatment (T0; baseline) and forty-two days (T42) after first injection (Figure S1). Induction of anesthesia was performed using 2% isoflurane (Merial) mixed with 100% oxygen (2 minutes) in an induction chamber. The mice were then placed on a heat pad in a supine position and kept at 37°C to minimize fluctuations of body temperature. Data acquisition was performed in mice lightly anesthetized with 0.5% to 1% isoflurane, in order to maintain heart rate ≥ 450 beats/min. Two-dimensional short-axis M-mode echocardiography was performed, and left ventricular end-systolic volume (LVSV), left ventricular end-diastolic volume (LVDV), and internal end-systolic and end-diastolic diameters (LVSD, LVDD) were measured; left ventricular ejection fraction (LVEF) and left ventricular fractional shortening (LVFS) were computed.

### 2.3. Evaluation of Plasma Cardiac Troponin and BNP

Cardiac troponin I was measured in plasma samples (*n* = 4/group) at T42 using the mouse cardiac Tn-I (high sensitivity) kit (Life Diagnostics), following the manufacturer's instructions. Plasma BNP was measured in plasma samples (*n* = 4/group) at T42 using the mouse BNP-EIA kit (RayBiotech), following the manufacturer's instructions.

### 2.4. Sample Collection

At sacrifice (T42), animals were anesthetized by CO_2_ overdose followed by cervical dislocation. Then, blood was collected into EDTA-coated tubes, immediately centrifuged to separate plasma as previously described [13], and stored at −80°C until further use.

### 2.5. Total RNA Purification

Total RNA purification from 200 *μ*L of plasma/sample was conducted using TRIzol (Life Technologies) following a modified protocol [14] for liquid specimens. RNA pellets were resuspended in RNAse-free water. Since RNA quantification from plasma is not possible, the samples were stored at −80°C until further use.

### 2.6. MicroRNA Screening

miRNA expression profiling was conducted at T42 using the TaqMan rodent microRNA A array v2.0 (Applied Biosystems), following the manufacturer's protocol for liquid specimens. The screening was performed on four plasma samples from each group. Data were analyzed using ExpressionSuite v1.0.3 dedicated software (Life Technologies), using the global normalization method, and all miRNAs presenting Ct values > 28 were considered as not expressed. All miRNAs showing a fold change ≥ 2 and a *p* value < 0.05 were selected for single-assay-based validation. Moreover, a few additional miRNAs were analysed, basing on their known involvement in cardiac diseases [13–15]: miR-1-3p, miR-133a-3p, and miR-499a-5p.

### 2.7. Single-miRNA Assays

MicroRNA retrotranscription was conducted using TaqMan Advanced miRNA cDNA synthesis kit (Life Technologies) starting from 2 *μ*L of total RNA. Expression levels of screening-selected miRNAs were evaluated using single TaqMan advanced miRNA assays (Life Technologies), following the manufacturer's protocol. Plasma miR-27-3p was selected as normalizer basing on screening results as previously described [16]. Indeed, it showed a strong expression in all samples with very limited intragroup variability when used for normalization. These observations were confirmed also by using the NormFinder (http://moma.dk/normfinder-software) software.

### 2.8. Statistical Analysis

A clustering analysis was performed, considering the assessed cardiac functional parameters (LVEF, LVFS, LVSD, LVDD, LVSV, and LVDV), in order to identify sample groups in an unsupervised fashion. The GENE-E software (http://www.broadinstitute.org/cancer/software/GENE-E/index.html) was used to draw heatmaps and dendograms. The dissimilarity matrix was computed on the basis of the Pearson's correlation. A Multidimensional scaling (MDS) in three dimensions was implemented to evaluate the discrimination power of selected differentially expressed miRNAs; the goodness of the grouping was evaluated by the average of the silhouette indexes (aSI) [17–19]. Differences in cardiac functional parameters was assessed by the Kruskal–Wallis test, (GraphPad Prism 5 software) while differences in miRNA expression level were evaluated by a two-way ANOVA, considering the treatment response and the time (T0 and T42) as sources of variability. The Tukey post hoc test was used to evaluate differences between each category pair. The “car,” “cluster,” and “stats” [20] R packages were exploited for this purpose. *p* values < 0.05 were considered statistically significant.

## 3. Results

### 3.1. Heterogeneous Cardiotoxic Effects of Doxorubicin on Mice

**Figure 1:**
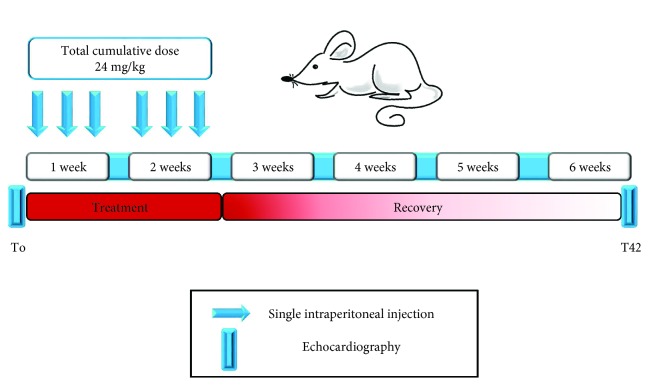
Experimental design. Treatment protocol for our animal model of DOX-induced cardiotoxicity. Arrows indicate single 4 mg/kg intraperitoneal DOX/saline injections. The horizontal blue bars indicate experimental endpoints when echocardiography was conducted. Each group was composed of ten animals.

**Figure 2:**
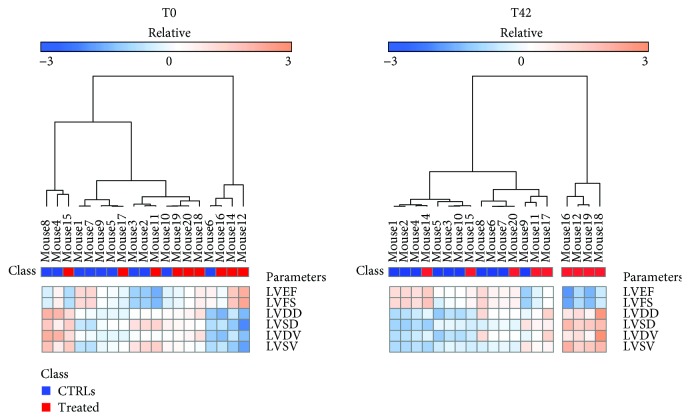
Differential cardiac response to doxorubicin treatment. Unsupervised clustering based on cardiac functional parameters at T0 (a) and T42 (b). At T0, all animals were grouped together. At T42, a cluster of drug-treated animals was clearly separated (Tox) from those unaffected (NoTox) by doxorubicin and CTRLs. Cardiac functional parameters: LVEF: left ventricular ejection fraction; LVFS: left ventricular fractional shortening; LVDD: left ventricular diastolic diameter; LVSD: left ventricular systolic diameter; LVDV: left ventricular diastolic volume; LVSV: left ventricular systolic volume.

**Figure 3:**
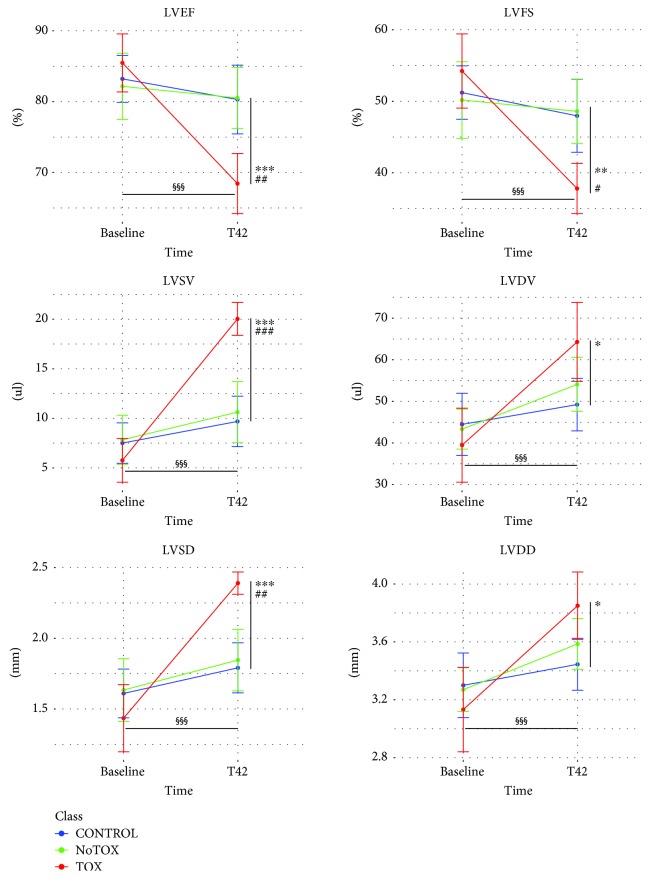
Cardiac dysfunction assessment. Cardiac parameter variation between baseline and T42 analyzed in each animal group: CTRL (blue), NoTox (green) and Tox (red). LVEF: left ventricular ejection fraction; LVFS: left ventricular fractional shortening; LVSV: left ventricular systolic volume; LVDV: left ventricular diastolic volume; LVSD: left ventricular internal systolic diameter; LVDD: left ventricular internal diastolic diameter. ^#^ *p* < 0.05, ^##^ *p* < 0.01, ^###^ *p* < 0.001 Tox vs. CTRL; ^∗^ = *p* < 0.05; ^∗∗^ = *p* < 0.01, ^∗∗∗^ = *p* < 0.001 Tox vs. NoTox; ^§§§^ = *p* < 0.001 Tox T42 vs. Tox T0. CTRL *n* = 10; NoTox *n* = 5; Tox *n* = 4.

In order to identify circulating miRNAs associated to adverse cardiac response to DOX, we assessed the onset of cardiotoxicity by cardiac echocardiography at baseline (T0) and 42 days (T42) after first injection (Figure 1). In particular, unsupervised clustering analysis of cardiac functional parameters indicated that some DOX-treated animals presented a heterogeneous response to drug administration at T42 (Figure 2). Indeed, basing on heart function data, five treated mice (NoTox) grouped with the CTRL animals, while a cluster of four mice (Tox) diverged from both CTRLs and NoTox. Of note, the same analysis, performed using baseline data, showed that all animals grouped together before DOX treatment, as confirmed by quantitative analysis of the six cardiac parameters. Differently, when considering the data obtained at T42, Tox mice showed impaired heart function vs. both NoTox and CTRL animals (Figure 3 and Supplementary Table 1). In particular, they presented a significant reduction in LVEF and LVFS, a strong increase in LVSV and LVDV, and a moderate rise in LVDD and LVDV. The comparison between T0 and T42 evidenced a detrimental effect of DOX only in the Tox group. Differently, CTRL and NoTox animals showed no appreciable variations in heart function throughout the duration of the experiment. Cardiac troponin I and BNP levels were measured as indicators of cardiac damage and dysfunction at T42. Interestingly, no variations from CTRL levels were observed for both markers in either NoTox or Tox animals (Figure S2).

### 3.2. Plasmatic miRNA Profiling and Validation at T42

**Figure 4:**
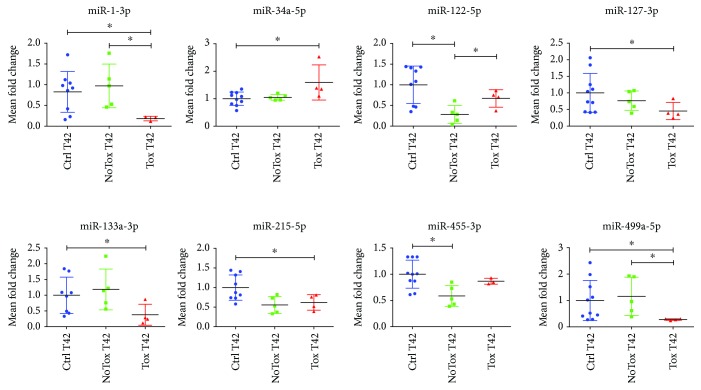
Plasma miRNAs are regulated upon doxorubicin treatment. Eight miRNAs showed dysregulated expression upon DOX treatment (vs. CTRL). Data are depicted as scatter plots and expressed as mean fold change ± SD vs. CTRL, arbitrarily set to 1. Controls: blue dots, NoTox: green dots, Tox: red dots. ^∗^ *p* < 0.05. CTRL *n* = 10; NoTox *n* = 5; Tox *n* = 4.

With the aim of identifying plasma miRNAs potentially associated to long-term post-DOX treatment, we conducted an array-based screening comparing CTRL, NoTox, and Tox samples at T42. We found fourteen miRNAs potentially regulated (*p* < 0.05) among all the groups (not shown). In particular, after validation, eight miRNAs evidenced a significant dysregulation in their plasma expression (Figure 4 and Supplementary Table 2). Interestingly, two miRNAs, miR-1-3p and miR-499a-5p, showed a strong decrease in the Tox group in comparison to both CTRLs and NoTox groups. Differently, miR-122-5p was decreased only in the NoTox group (when compared to CTRLs and Tox mice), similarly to miR-455-3-p (vs. CTRLs only). In addition, miR-127-3p, miR-133a-3p, and miR-215-5p showed a significant downregulation in Tox vs. the CTRL groups, while miR-34a-5p was upregulated.

### 3.3. Circulating miRNA-Based Distinction of DOX-Affected from DOX-Unaffected Mice

**Figure 5:**
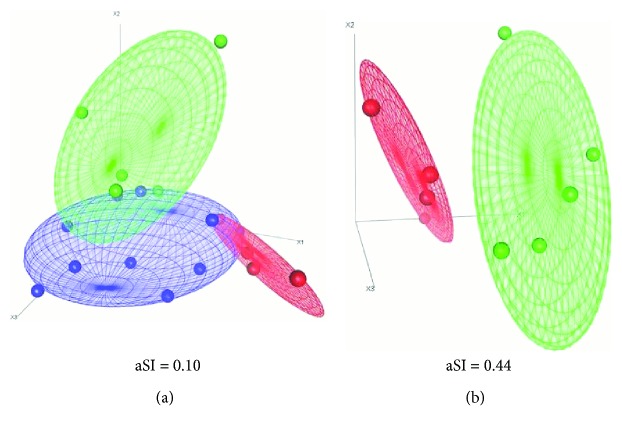
Plasma miRNAs can be used to identify doxorubicin response in treated animals. Multidimensional scaling analysis was used to investigate whether the expression of regulated plasma miRNAs could be used to correctly identify treated animals showing different cardiac responses to doxorubicin. (a) All Tox miRNAs were used for unsupervised clustering. A good accuracy was observed in separating the three groups of animals. (b) We identified a restricted miRNA cluster (miR-1-3p, miR-34a-5p, miR-133a-3p, and miR-499a-5p) correctly separating Tox/NoTox mice. Blue: CTRL; green: NoTox; red: Tox. aSI: average silhouette index. CTRL *n* = 10; NoTox *n* = 5; Tox *n* = 4.

These results prompted us to investigate whether DOX-regulated miRNAs could be used to distinguish drug-affected from drug-unaffected animals. Thus, we performed a Multidimensional scaling analysis basing on the expression of all eight regulated miRNAs. As shown in Figure 5(a), this miRNA signature correctly discriminated CTRLs from NoTox and Tox animals, as indicated by the calculated average silhouette indexes (0.10). Then, we conducted the same analysis only on DOX animals, in order to identify those miRNAs with the best discriminatory potential between Tox vs. NoTox mice. We identified a cluster composed of four miRNAs (miR-1-3p, miR-34a-5p, miR-133a-3p, and miR-499a-5p) with the better discriminatory potential between the two groups (aSI: 0.44). Indeed, a complete partition could be observed between DOX-affected and DOX-unaffected mice (Figure 5(b)).

## 4. Discussion

This is the first study showing that, similarly to cancer patients, animal models do not homogeneously respond to anthracycline treatment. The main finding of this work is that circulating miRNAs discriminate drug-affected from drug-unaffected animals after a long-term doxorubicin exposure. In clinical practice, the most important DOX-related issue relies in the timely detection of cardiac complications in treated patients, usually occurring even years after administration [21]. The correct identification of subjects at high risk of cardiac dysfunction onset is, in fact, of utmost importance to ensure appropriate interventions [11]. In our murine model, cardiotoxicity was induced by serial injections of doxorubicin. Interestingly, the presence of heart dysfunction, although suggested by the alteration of some cardiac parameters, was not clearly evident in long-term recovering animals. Thus, differently from previous works [22], we decided to perform a thorough evaluation of cardiac parameters in order to assess the real effects of the drug on treated animals. Surprisingly, our analysis evidenced a significant difference of cardiac response to DOX administration among treated mice. Indeed, we identified a cluster of animals displaying either none or negligible functional defects (NoTox), while another group showed a strong cardiac impairment due to the drug (Tox). Consistent with this observation, our array-based screening led to the identification of several regulated plasma miRNAs both in Tox and NoTox animals in comparison to controls. Interestingly, we assessed a DOX-induced upregulation of miR-34a-5p only in plasma of Tox animals. This result is in line with a previous work indicating a miR-34a-5p increase at circulating levels [11] upon acute anthracycline administration, although we are the first to report its regulation after a long-term release from treatment. Intriguingly, beside DOX-induced cardiotoxicity [23], increased circulating levels of miR-34a-5p have been associated with ventricular remodelling [24] and heart failure onset [25, 26] following myocardial infarction. Moreover, its inhibition seems to have beneficial effects against cardiac dysfunction [27], hinting at its possible clinical exploitation both as a biomarker and therapeutic target.

An interesting finding was represented by the observed dysregulation, in the Tox group, of miR-1-3p, miR-133a-3p, and miR-499a-5p, previously observed to be increased in plasma of patients suffering from cardiac damage [28]. In particular, the circulating expression of the latter has previously been associated to plasma troponin levels during infarction [29]. Surprisingly, though, these three miRNAs showed a strong decrease in Tox animals at T42 when compared to CTRL and NoTox animals, possibly because of a response mechanism triggered by treatment. Noteworthy, during our experiments, we did not observe any perturbation in plasma levels of cardiac troponin I and BNP, both considered as the most important markers of cardiotoxicity [30, 31]. These results, though, seem not to be an uncommon event for *in vivo* studies [32], and we cannot exclude that technical limitations somehow influenced the results.

Among the other regulated miRNAs, only miR-215-5p was previously shown to be “positively” perturbed upon chronic doxorubicin treatment, although it was found upregulated in rat hearts at different times and amount of administered drug [33]. In regard to miR-127-3p, its role in heart physiology and cardiotoxicity has never been assessed, although it was found downregulated in patients suffering from acute pancreatitis associated with lung injury [14, 34]. Interestingly, both miR-122-5p and miR-455-3p showed a differential expression only in the NoTox group, again possibly because of some unknown effects triggered by heart dysfunction in Tox animals. Of note, plasma miR-122-5p, despite being known as liver-specific, has been already reported to be negatively regulated in cardiovascular diseases [35]. As for miR-455-3p, it was demonstrated to be an enhancer for hypertrophy in a murine model, but, at the same time, it reduced the progressive deterioration of left ventricular function [36, 37].

The principal novelty of this work relies on the identification of a miRNA signature that separates with good efficiency drug-affected both from drug-unaffected and control mice. Indeed, the whole set of DOX-regulated miRNAs showed a good accuracy in separating the three “functional” groups of animals. Our analyses, though, showed that not all DOX-regulated miRNAs were necessary to correctly separate the groups of animals. Notably, a subsignature composed of four out of eight circulating miRNAs regulated by DOX showed to be sufficient to correctly identify Tox from NoTox animals.

Some limitations of this work should be acknowledged. Since we used healthy animals, tumor contribution to the plasmatic composition of miRNAs and possible detrimental systemic effects of cancer could not be assessed. Indeed, the expression of some circulating miRNAs, including miR-34a-5p, is known to be affected by some tumors [38], but we decided to focus our attention on cardiac effects of the drug in a “clean environment.” Nevertheless, our results partially overlap (at least for miR-1-3p) with those from recent investigations conducted on cancer patients [14], although we are aware that the time frame evaluated in our animal model is not comparable to those evaluated in clinical studies. We are aware that by focusing only on female mice, we partly reduced the strength of our findings. Nevertheless, since our aim was mimicking female breast cancer patients, we feel that including male subjects in our study could result in the unwanted addition of possible confounding variables. Since oestrous cycle was not evaluated, a possible influence on the cardiotoxic process described here cannot be excluded. Female reproductive hormones have been proposed to be associated with cardioprotective states; however, although several mechanisms have been suggested for the possible underlying mechanism, no study elucidated the potential changes of the cardiac tissue during specific stages of the oestrous cycle. Evaluating the influence of fluctuating oestrous cycle hormones on cardiac tissue remodelling was out of our aim, and we believe that this topic deserve a specific investigation. In addition, beside baseline and long-term data, we did not evaluate miRNAs and functional cardiac parameters in the acute phase of treatment. Indeed, we decided to focus on the follow-up period, usually the most critical time for cardiotoxicity onset, and the one never previously investigated in animal models. Further, patient-based investigations are needed to verify whether our findings can be applied to the clinical setting. Anyhow, we demonstrated, for the first time, the existence of a heterogeneous cardiac functional response to DOX over time, which is reflected by variation of expression of specific clusters of circulating miRNAs.

## Data Availability

The screenings datasets generated and analyzed during the current study are available from the corresponding authors on request.

## Supplementary Materials

Supplementary 1 Supplementary Figure S1: echocardiographic imaging. The panel depicts three representative two-dimensional short-axis echocardiographic images (M-mode) of CTRL, NoTox and Tox groups at T0 (left) and T42 (right). No differences were observed among the groups at T0, while the Tox group showed signs of dysfunction at T42.

Supplementary 2 Supplementary Figure S2: plasma troponin I and BNP levels. Plasma TnI and BNP levels were measured in CTRL (blue bars) and NoTox (green bars) and Tox (red bars) animals (*n* = 4/group) at T42. No differences were observed among any group. Values are expressed as mean ± SD.

Supplementary 3 Supplementary Table S1: cardiac function parameters of treated and control animals and *p* values of performed comparisons. Parameter values are expressed as mean ± SD.

Supplementary 4 Supplementary Table S2: fold change data of regulated miRNAs. Values are expressed as mean ± SD.
