# Supplementary figures and images for: A Specific Circulating MicroRNA Cluster Is Associated to Late Differential Cardiac Response to Doxorubicin-Induced Cardiotoxicity In Vivo
Source: Dis Markers. 2018 Dec 9;2018:8395651. doi: 10.1155/2018/8395651 (PMC6304816; doi:10.1155/2018/8395651)

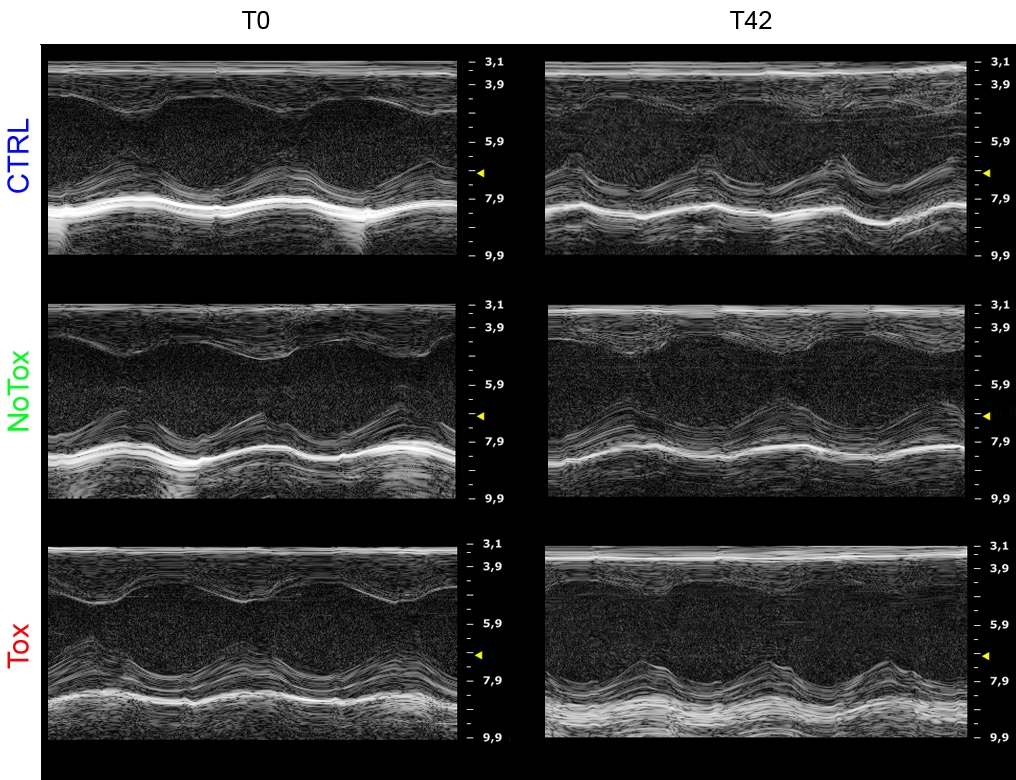

Supplement: Supplementary 1 — Supplementary Figure S1: echocardiographic imaging. The panel depicts three representative two-dimensional short-axis echocardiographic images (M-mode) of CTRL, NoTox and Tox groups at T0 (left) and T42 (right). No differences were observed among the groups at T0, while the Tox group showed signs of dysfunction at T42. [file 8395651.f1.jpg]

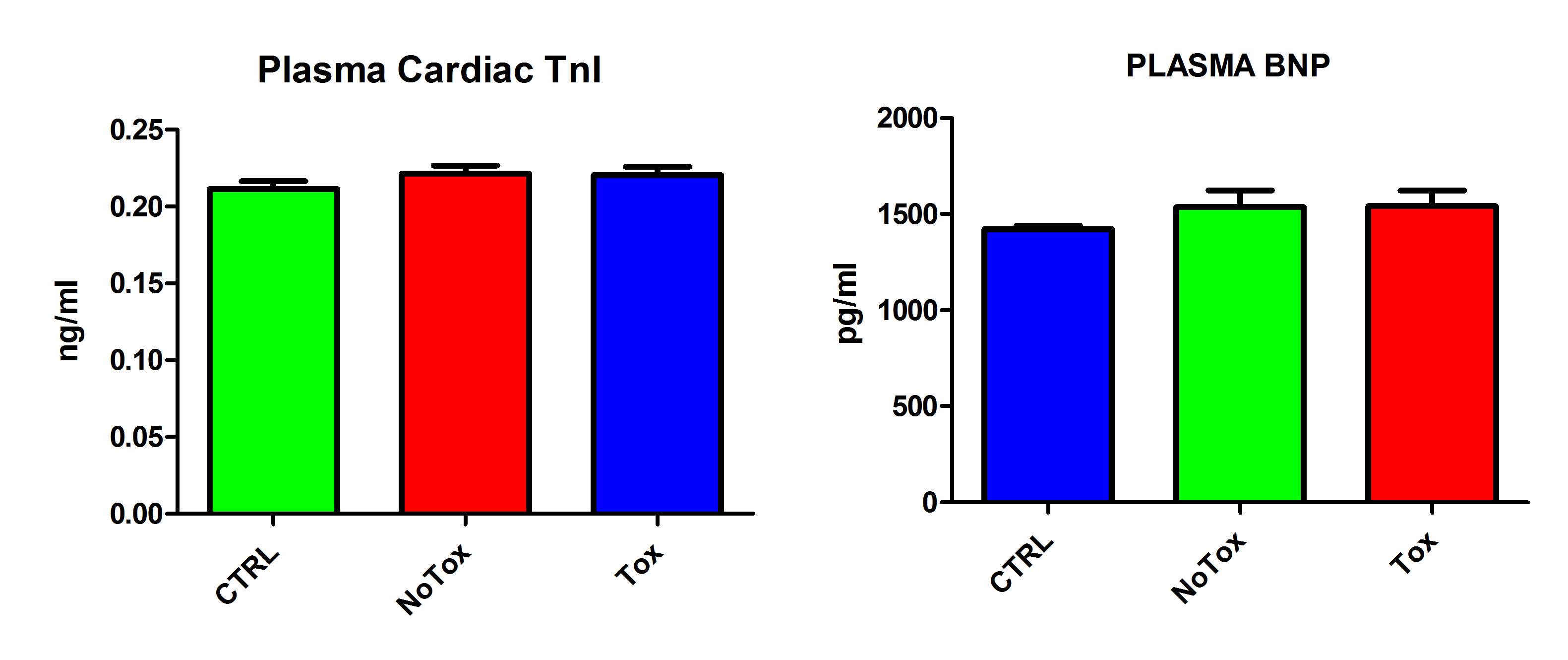

Supplement: Supplementary 2 — Supplementary Figure S2: plasma troponin I and BNP levels. Plasma TnI and BNP levels were measured in CTRL (blue bars) and NoTox (green bars) and Tox (red bars) animals (n = 4/group) at T42. No differences were observed among any group. Values are expressed as mean ± SD. [file 8395651.f2.jpg]
